# Reasons for accepting or declining to participate in randomized clinical trials for cancer therapy

**DOI:** 10.1054/bjoc.2000.1142

**Published:** 2000-06

**Authors:** V Jenkins, L Fallowfield

**Affiliations:** CRC Psychosocial Oncology Group, Royal Free and University College London Medical School, 3rd Floor Bland Sutton Institute, 48 Riding House Street, London, W1P 7PL, UK

**Keywords:** cancer, randomized clinical trials, communication, trial participation

## Abstract

This paper reports on the reasons why patients agreed to or declined entry into randomized trials of cancer following discussions conducted by clinicians in both District General and University Hospitals. Two hundred and four patients completed a 16-item questionnaire following the consultation, of these 112 (55%) were women with breast cancer. Overall results showed that 147 (72.1%) patients accepted entry to a randomized clinical trial (RCT). The main reasons nominated for participating in a trial were that ‘others will benefit’ (23.1%) and ‘trust in the doctor’ (21.1%). One of the main reasons for declining trial entry was that patients were ‘worried about randomization’ (19.6%). There was a significantly higher acceptance rate for trials providing active treatment in every arm 98 (80.6%) compared with those trials with a no treatment arm 46 (60.5%), χ^2^test *P* = 0.003. The study outlines a number of factors that appear to influence a patient’s decision to accept or decline entry into an RCT of cancer therapy. An important factor is whether or not the trial offers active treatment in all arms of the study. Communication that promotes trust and confidence in the doctor is also a powerful motivating influence. © 2000 Cancer Research Campaign


*British Journal of Cancer* (2000) **82**(11), 1783–1788
© 2000 Cancer Research Campaign
DOI: 10.1054/ bjoc.2000.1142, available online at http://www.idealibrary.com on IDEAL®

### Reasons for accepting or declining to participate in randomized clinical trials for cancer therapy

**V Jenkins and L Fallowfield**

CRC Psychosocial Oncology Group, Royal Free and University College London Medical School, 3rd Floor Bland Sutton Institute, 48 Riding House Street, London W1P 7PL, UK

**Summary** This paper reports on the reasons why patients agreed to or declined entry into randomized trials of cancer following discussions conducted by clinicians in both District General and University Hospitals. Two hundred and four patients completed a 16-item questionnaire following the consultation, of these 112 (55%) were women with breast cancer. Overall results showed that 147 (72.1%) patients accepted entry to a randomized clinical trial (RCT). The main reasons nominated for participating in a trial were that 'others will benefit' (23.1%) and 'trust in the doctor' (21.1%). One of the main reasons for declining trial entry was that patients were 'worried about randomization' (19.6%). There was a significantly higher acceptance rate for trials providing active treatment in every arm 98 (80.6%) compared with those trials with a no treatment arm 46 (60.5%), $\chi^2$ test $P = 0.003$. The study outlines a number of factors that appear to influence a patient's decision to accept or decline entry into an RCT of cancer therapy. An important factor is whether or not the trial offers active treatment in all arms of the study. Communication that promotes trust and confidence in the doctor is also a powerful motivating influence. © 2000 Cancer Research Campaign

**Keywords**: cancer; randomized clinical trials; communication; trial participation

Many patients with cancer have benefited from the introduction of new drug and treatment regimens. This is reflected both in an increase in survival and improved quality of life of patients undergoing different cancer treatments (Hamilton, 1992). As practise becomes more evidence-based, the medical profession and governmental agencies recognize that the most accurate way of evaluating new treatments is within a randomized clinical trial (RCT) (Smyth et al, 1994). Unfortunately, despite the fact that many UK clinicians are committed to the concept of a trial, fewer than 5% of UK patients are recruited, although the figure varies according to the cancer site and treatment centre (Stenning, 1992; Leonard, 1997).

Studies examining the reason for low recruitment note that non-participation is influenced by factors affecting both the physician and the patient, as well as the eligibility criteria in strict trial protocols (Taylor and Kelner, 1987; Cook-Gotay, 1991; Fallowfield et al, 1997). The UK Co-ordinating Committee on Cancer Research working party identified several factors affecting clinician participation, particularly for those doctors practising in non-teaching hospitals. Among these were time constraints and lack of support staff to help discuss and coordinate the pragmatic aspects of randomized trials (Slevin et al, 1995; Smyth et al, 1994).

Many patients presume that the cancer specialist will know exactly how to treat the illness. They will invest trust in the specialist, even before meeting them, and would not anticipate a consultation in which uncertainty and randomization are discussed. The notion of random assignment may be contrary to how a patient may view the delivery of cancer treatment (Toynbee, 1997). In addition, the patient may interpret the word 'trial' to mean that they are to take part in an experiment, never before tested on humans. One way to help overcome this belief is by educating the 'well' public about the necessity and positive aspects of trials (Baum, 1993; Saunders et al, 1994). The need for such action is supported in a recent study that explored the attitudes of 20 women with breast cancer to trials (Ellis and Butow, 1998). The women did not have a good understanding of the necessity for trials, nor did they understand the need for randomization of treatments.

A number of studies have examined the problem of informed consent and trials but few have focused on the reasons why patients with cancer accept, or decline, trial entry. Among the factors that have been identified for acceptance of trial participation are the hopes that the new treatment will be of benefit to them, and that it will be of benefit to others (Penman et al, 1984; Kardinal, 1994; Slevin et al, 1995). Reasons for declining participation include a preference for a specific treatment arm and fear of randomization (Penman et al, 1984; Llewellyn-Thomas et al, 1991; Jenkins et al, 1999). This paper examines some of the reasons given by patients for accepting or declining entry to different types of randomized trials of cancer therapy and is part of a study being conducted in the UK that aims to improve communication between clinicians and patients when trials are discussed.

### MATERIALS AND METHODS

### Questionnaire

The questionnaire used was a similar design to that developed by Penman and colleagues (Penman et al, 1984) and piloted on 50 patients with cancer who had agreed to participate in cancer trials.

*Received 4 August 1999*
*Revised 7 February 2000*
*Accepted 17 February 2000*

*Correspondence to:* V Jenkins

The revised version of the questionnaire was administered following the discussion of randomized trials with the clinician and completed by the patient at home. The layout of the questionnaire is shown in Appendix 1. First, patients indicated whether or not they had agreed to take part in a randomized trial or if they did not know. Next the questionnaire listed 16 possible reasons that might have influenced the decision to either accept or decline treatment. For each statement patients registered their agreement or disagreement on a scale of 1 (strongly agree) to 5 (strongly disagree). Finally, patients indicated the most important reason for their decision to accept or decline to take part in the trial.

### Sample

Two hundred and forty patients with cancer and eligible to participate in randomized clinical trials were invited to join the main study. They comprised newly diagnosed and relapsed patients referred to 17 senior clinicians (three specialist breast surgeons, five medical oncologists and nine clinical oncologists) at district general and university teaching hospitals. Nineteen patients declined to participate in the communication study and, of the 221 who took part, 204 (92.3%) returned the questionnaires. Tables 1 and 2 show the characteristics of these patients, with breast cancer patients forming 55% of the total sample. The high percentage of patients with breast cancer probably reflects the large numbers of trials currently being conducted in this common tumour site. Few patients had previous trial experience (11/204, 5.4%) or previous experience of chemotherapy (17/204, 8.3%). Only nine patients were expecting to discuss trials with the clinician during the consultation.

### Method

The design and method for the main study have been described elsewhere in detail (see Jenkins et al, 1999). The study had the approval of the Trent Multi Regional Ethic Committee and the Local Ethic Committees of the participating hospitals. Before the consultation, patients completed three questionnaires: a Patient Information Needs questionnaire, a Patients' Attitudes to Randomised Clinical Trials questionnaire and the Speilberger State Trait Anxiety Inventory (STAI). The consultations were audiotaped and the patient was subsequently given two further questionnaires to complete and return by post. One of the questionnaires examined patients' satisfaction with the consultation and the other reasons for accepting or declining to enter a clinical trial. During the consultation the clinician discussed the randomized clinical trial (RCT) suitable for that patient (e.g. ATAC, QASAR etc.) and in some cases a research nurse was present to provide additional information. In addition, most patients received information sheets about the treatment trial either during or following the consultation. The data presented in this paper are from the postal questionnaire (Appendix 1), examining the reasons why patients agreed or declined to participate in RCTs and whether the decision was influenced by the type of trial on offer. The data were analysed using a standard SPSS package. A report of the findings from the main study will be available later in the year.

**Table 1** Age and sex distribution (*n* = 204)

| Age range | Male *n* (%) | Female *n* (%) |
|---|---|---|
| >25 years | 3 (1.5) | |
| 25–44 years | 9 (4.4) | 19 (9.3) |
| 45–64 years | 22 (10.8) | 84 (41.2) |
| over 65 years | 28 (13.7) | 39 (19.1). |

**Table 2** Cancer site distribution (*n* = 204)

| Cancer site | *n* (%) |
|---|---|
| Breast | 112 (55) |
| Prostate | 23 (11) |
| Testicular | 15 (7) |
| Lung | 7 (3) |
| Colorectal | 15 (7) |
| Ovarian | 18 (9) |
| Melanoma | 2 (1) |
| Lymphoma | 2 (1) |
| Bladder | 4 (2) |
| Pancreas | 5 (2.5) |
| Brain | 1 (0.5) |

### RESULTS

Overall, 147/204 (72.1%) patients accepted entry to a clinical trial, 51/204 (25%) declined and 6/204 (2.9%) indicated that they did not know. This uncertainty may refer to the fact that they were still undecided or that they were unsure whether they had agreed to participate in a trial. Information sheets were given to 157/204 (77%) patients about the trial during the consultation. Of the 47 patients who did not receive an information sheet, 29/47 (64.4%) agreed to participate in a trial and 16/47 (35.6%) declined. In addition, a third of patients 68/204 (33.3%) had extra information provided to them by a research nurse or trial coordinator and, of these, 55/68 (80%) agreed to participate and 13/68 (19.1%) declined. The number of patients with previous trial experience was 11 (5.4%), and of these only one declined and 15/17 (88.2%) patients who had previous experience of chemotherapy took part in a trial. There were no differences between those that accepted or declined trial entry according to marital status, age or level of anxiety.

### Reasons for accepting or declining trial entry

Table 3 displays the frequency (expressed as percentage) of agreement to each statement according to whether patients accepted or declined trial entry. The categories 'strongly agree' and 'agree to some extent' were combined and differences between the groups analysed using the Mann–Whitney test for non-parametric data. Table 4 shows the most important reason given by patients for deciding to accept or decline trial entry. The results shown in the following tables exclude the six patients who did not know whether they were in a trial or not.

Altruism and trust in the doctor are seen as the most important reasons for accepting entry to a trial, whereas preference for the doctor choosing treatment rather than randomization are cited for declining a trial. A total of 35 different kinds of trial were discussed and because of the small numbers in some of the trials they were divided into four broad categories for analysis:

1. Chemotherapy
2. Radiotherapy
3. Hormone therapy
4. Miscellaneous.

*© 2000 Cancer Research Campaign*

**Table 3** The frequency (expressed as percentage) of agreement to each statement according to whether patients accepted or declined trial entry.

| | Statement | Accept trial (*n* = 147) | Decline trial (*n* = 51) | *P*-value |
|---|---|---|---|---|
| 1. | I thought the trial offered the best treatment available | 82.3% (121) | 11.8% (6) | 0.0001 |
| 2. | I believed the benefits of treatment in the trial would outweigh the side-effects | 78.9% (116) | 11.8% (6) | 0.0001 |
| 3. | I was satisfied that either treatment in the trial would be suitable | 81% (119) | 13.7% (7) | 0.0001 |
| 4. | I was worried that my illness would get worse unless I joined the trial | 17% (25) | 9.8% (5) | 0.24 |
| 5. | The idea of randomization worried me | 38.1% (56) | 62.7% (32) | 0.049 |
| 6. | I wanted the doctor to choose my treatment rather than be randomized by computer | 51% (75) | 76.5% (39) | 0.0039 |
| 7. | The doctor told me what I needed to know about the trial | 95.9% (141) | 88.2% (45) | 0.0553 |
| 8. | I trusted the doctor treating me | 97.3% (143) | 94.1% (48) | 0.2935 |
| 9. | I was given too much information to read about the trial | 7.5% (11) | 7.8% (11) | 0.0982 |
| 10. | I was given enough information to read about the trial | 81.6% (120) | 56.9% (29) | 0.0003 |
| 11. | I knew I could leave the trial at any time and still be treated | 97.3% (143) | 90.2% (46) | 0.0345 |
| 12. | I did not feel able to say no | 10.2% (15) | 15.7% (8) | 0.1039 |
| 13. | I wanted to help with the doctor's research | 92.5% (136) | 45.1% (23) | 0.0001 |
| 14. | I feel that others with my illness will benefit from the results of the trial | 97.3% (143) | 58.8% (30) | 0.0001 |
| 15. | The doctor wanted me to join the trial | 52.4% (77) | 31.4% (16) | 0.0144 |
| 16. | Others, e.g. family or friends wanted me to join the trial | 43.5% (64) | 3.9% (2) | 0.0002 |

**Table 4** Values are numbers (percentage) of patients

| | |
|---|---|
| Top reasons for accepting trial entry *n* = 138 (nine missing cases) | *n* (%) |
| I feel that others with my illness will benefit from the results of the trial | 34 (23.1) |
| I trusted the doctor treating me | 31 (21.1) |
| I thought the trial offered the best treatment available | 24 (16.3) |
| Top reasons for declining trial entry *n* = 47 (four missing cases) | *n* (%) |
| I trusted the doctor treating me | 11 (21.6) |
| The idea of randomization worried me | 10 (19.6) |
| I wanted the doctor to choose my treatment rather than be randomized by computer | 9 (17.6) |

The participation rates for these categories are shown in Table 5. There was a lower rate of acceptance for the chemotherapy and radiotherapy trials compared with the hormone treatment trials. This was not necessarily due to the relative acceptability of the different treatments per se, but because of the trial design. Some trials involved a placebo and others a 'no treatment' arm. A further analysis was performed to examine this issue, with trials broadly classified into three categories, those with:

1. Two or more active treatment arms
2. A 'no treatment arm'
3. A placebo arm.

The acceptance rates for these categories are shown in Table 6. There was a significantly higher acceptance rate for trials with an active treatment arm, 79/98 (80.6%) compared with those with no treatment arm 46/76 (60.5%), $\chi^2$ test, $P$ = 0.003. Surprisingly the

**Table 5** Values are numbers (%) of patients

| Trial category | n | Accept | Decline |
|---|---|---|---|
| Chemotherapy | 90 | 60 (66.7%) | 30 (33.3%) |
| Radiotherapy | 25 | 15 (60%) | 10 (40%) |
| Hormone therapy | 76 | 65 (85.5%) | 11 (14.5%) |
| Miscellaneous | 7 | 7 (100%) | |

**Table 6** Values are numbers (%) of patients

| Trial category | n | Accept | Decline |
|---|---|---|---|
| Active treatment arms | 98 | 79 (80.6) | 19 (19.4) |
| No treatment arm | 76 | 46 (60.5) | 30 (39.5) |
| Placebo arm | 24 | 22 (91.7) | 2 (8.3) |

rate of acceptance to the placebo trials was high, but it should be noted that 19/22 (86.3%) of the patients were all offered the same trial for prostate cancer by one female clinician.

### DISCUSSION

The results show that the majority of patients offered entry into a trial accepted. The main reason for participating was that the treatment would benefit others in the future. However, this reason was closely followed by trust in the doctor. The importance of altruism as a motivating factor complements previous studies that examined patients considering hypothetical and non-cancer treatment trials (Mattson et al, 1985; Welton et al, 1999). Altruism is also cited as a motivating factor for participating in phase I cancer trials where there is no long-term benefit for the patient (Kardinal, 1994). Whilst it is possible that patients with cancer are selfless, one must also consider such concepts as social desirability. Social desirability depends on a number of factors including sex, cultural background and the specific question asked. If patients believe another will read their responses, in order to be viewed in a good light, they may give a socially desirable response (Streiner and Norman, 1989).

'Trust in the doctor' was the second most frequently endorsed reason for joining a cancer trial. This finding has remained stable for over a decade and forms an important part of the doctor–patient relationship (Penman et al, 1984). The provision of faith and hope is seen as a central feature of a 'healing' relationship and are powerful agents in their own right. Patients with cancer are faced with a life-threatening illness and invest a lot of faith in the doctor. Lupton (1996) notes that although patients judged the doctor's medical knowledge to be an important feature it was not the essential characteristic that made a 'good' doctor. The key features considered by the majority of patients were trust and interpersonal skills, especially listening and communication.

Remarkably, 'trust in the doctor' was also cited as the main reason for declining to participate in a trial. In the absence of detailed interviews the patients' interpretation of this phrase is unclear. Perhaps during the discussion patients felt that the doctor was unenthusiastic about the trial. Llewellyn-Thomas and colleagues (Llewellyn-Thomas et al, 1991) suggested that cancer patients who agree to enter clinical trials might be more susceptible to the clinicians' enthusiasm for the trial. Alternatively, the clinician may have provided the patient with an unbiased, objective view stressing the voluntary nature of the study. Patients may have over interpreted this equipoise to mean that 'standard treatment' was better than the experimental arm and would not compromise survival. There is evidence that cancer patients sometimes overestimate the benefits of standard therapies (Sheldon et al, 1993). Other reasons given for declining to participate in a trial were a fear of randomization and preference for the doctor to choose the treatment. These two reasons combined related to the same issue – the dislike of the idea that the choice of treatment would be based on chance. What one does not know is whether patients declined because they **did not** understand the concept of randomization or because they **did** understand the concept. Previous studies would suggest it is the former reason combined with either a poor explanation of the concept by the doctor, or too explicit an explanation (Corbett et al, 1996). The preference for the doctor choosing the treatment was not exclusive to the decliners. Fifty-one per cent of those who agreed to a trial indicated that they would have preferred the doctor to choose the treatment. This was reported as one of the less appealing aspects of randomized trials in previous research (Slevin et al, 1995). The emphasis given to chance in the explanation of the concept of randomization is another cause of unease amongst patients and the general public (Corbett et al, 1996; Fallowfield et al, 1998; Featherstone and Donovan, 1998).

The differences between those who accepted and those who declined trial entry are shown by their response to the statements in the questionnaire. Those who decided to participate in a trial agreed more with the statements that emphasized the benefits of treatment, and were worried that their illness would get worse if they did not join. They appeared to be more influenced by the doctor, family and friends than those who declined and, furthermore, agreed that they wanted to help with the doctor's research.

Those who declined were somewhat less satisfied with the amount of written information given to them about the trial, but there was no statistical difference between the groups. The decliners had more reservation about the treatment being randomized and did not agree that the trial offered the best available treatment.

Although the subgroups were unequal, the acceptance rates to the trials differed according to the type of treatment and type of trial. The highest acceptance rate was recorded for the hormone trials of different treatments compared with the chemotherapy and radiotherapy studies. Perhaps patients view chemotherapy and radiotherapy as short-term intensive treatments, which are more intrusive and time-consuming. Additionally, clinicians may understate the potential severity of hormone treatment. Side-effects associated with hormone therapy have not been the subject of systematic evaluation to the same extent as chemotherapy (Leonard et al, 1996), whereas discussion of 'serious' side-effects such as nausea, vomiting and hair loss are talked about more frequently for chemotherapy treatments (Jenkins et al, 1999). When the trials were categorized according to trial design, significantly more patients declined entry to a trial with no treatment arm. This type of study is often the most controversial and of course the discussion of these trials often poses the biggest problem for doctors (Cook-Gotay, 1991).

It was somewhat surprising to find that 23% of patients were not provided with a written information leaflet despite the fact that this is a mandatory requirement for ethics approval of any RCT, and

 © 2000 Cancer Research Campaign that the clinicians knew that their consenting procedures were being scrutinized during the study. The results from the study show that patients are generally very willing to participate in studies but that type of trial and probably communication style of doctor or nurse explaining the study exerts a considerable influence on patients' preparedness to accept or decline.

### ACKNOWLEDGEMENTS

The authors would like to thank Professor Robert Souhami who commented on early drafts of the paper, the clinicians at University College London Hospitals, Southend, Hillingdon, Worthing and Brighton District General Hospitals for participating in the study and especially the patients. This work is part of a project funded by the NHS R&D programme. Lesley Fallowfield is supported by the Cancer Research Campaign.

### REFERENCES

Baum M (1993) Clinical trials are ethically impossible. *Lancet* **341**: 812–813

Cook Gotay C (1991) Accrual to cancer clinical trials: directions from the research literature. *Soc Sci Med* **33**: 569–577

Corbett F, Oldham J and Lilford R (1996) Offering patients entry in clinical trials: preliminary study of the views of prospective participants. *J Med Ethics* **22**: 227–231

Ellis PM and Butow P (1998) Focus group interviews examining attitudes to randomised trials among breast cancer patients and the general community. *Aust NZ J Public Health* **22**: 528–531

Fallowfield LJ, Ratcliffe D and Souhami RL (1997) Clinicians' attitudes to clinical trials of cancer therapy. *Eur J Cancer* **33**: 2221–2229

Fallowfield LJ, Jenkins V, Brennan C, Sawtell M, Moynihan C and Souhami RL (1998) Attitudes of patients to clinical trials of cancer therapy. *Eur J Cancer* **34**: 1554–1559

Featherstone K and Donovan J (1999) Random allocation or allocation at random? Patients' perspectives of participation in an RCT. *Br Med J* **317**: 1177–1180

Hamilton C (1992) Ethical and practical problems in trials testing treatment for pre-malignant conditions: breast cancer as a model. In: *Introducing New Treatments for Cancer. Practical, Ethical and Legal Problems*, Williams CJ (ed), pp. 315–322. Wiley, London

Jenkins VA, Fallowfield LJ, Souhami A and Sawtell M (1999) How do doctors explain randomised clinical trials to their patients? *Eur J Cancer* **35**: 1187–1193

Kardinal CG (1994) Ethical issues in cancer clinical trials. *J LA State Med Soc* **146**: 359–361

Leonard RC (1997) The advancement of high dose chemotherapy and dose intensification schedules. *Ann-Oncol* **8** (Suppl 3): S3–6

Leonard RCF, Lee L and Harrison ME (1996) Impact of side-effects associated with endocrine treatments for advanced breast cancer: clinicians' and patients' perceptions. *Breast* **5**: 259–264

Llewellyn-Thomas HA, McGreal MJ, Thiel EC, Fine S and Erlichman C (1991) Patients' willingness to enter clinical trials: measuring the association with perceived benefits and preference for decision participation. *Soc Sci and Med* **32**: 35–42

Lupton D (1996) Your life in their hands: trust in the medical encounter. In: *Health and the Sociology of Emotions*, James V and Gabe J (eds), pp. 157–172. Blackwell Press, Edinburgh

Mattson ME, Curb DJ, McArdle R and the Aspirin Myocardial Infarction Study and Beta Blocker Heart Attack Trial Research Groups (1985) Participation in a clinical trial: the patients' point of view. *Controlled Clin Trials* **6**: 156–167

Penman DT, Holland JC, Bahna GF, and Morrow G et al (1984) Informed consent for investigational chemotherapy: patients' and physicians' perceptions. *J Clin Oncol* **2**: 849–855

Saunders CM, Baum M and Houghton J (1994) Consent, research and the doctor–patient relationship. In: Gillon R (Ed) Principles of Health Care Ethics.

Saunders C (1996) Clinical trials: ethical, legal and practical considerations. In: *Medico-legal Essentials in Healthcare*, Payne-James J, Dean P and Wall I (eds), pp. 161–169. Churchill Livingstone, Edinburgh

Sheldon JM, Fetting JH and Siminoff LA (1993) Offering the option of randomised clinical trials to cancer patients who overestimate their prognoses with standard therapies. *Cancer Invest* **11**: 57–62

Slevin M, Mossman J, Bowling A, Leonard R, Steward W, Harper P, Mclllmurray M and Thatcher N (1995) Volunteers or victims: patients' views of randomised cancer clinical trials. *Br J Cancer* **71**: 1270–1274

Smyth JF, Mossman J, Hall R, Hepburn S, Pinkerton R, Richards M, Thatcher N and Box J (1994) Conducting clinical research in the new NHS. *Br Med J* **309**: 457–461

Stenning S (1992) The 'uncertainty principle': selection of patients for cancer clinical trials. In: *Introducing New Treatments for Cancer. Practical, Ethical and Legal Problems*, Williams CJ (ed), pp. 161–172. Wiley, London

Streiner DL and Norman GR (1989) *Health Measurement Scales. A Practical Guide*. Oxford Medical Press, Oxford

Taylor KM and Kelner M (1987) Interpreting physician participation in randomised clinical trials: the physician orientation profile. *J Health Soc Beh* **28**: 389–400

Toynbee P (1997) Random clinical trials are one of life's biggest gambles. *BMA News Rev* **50**: 50

Welton AJ, Vickers MR, Cooper JA, Meade TW and Marteau TM (1999) Is recruitment more difficult with a placebo arm in randomised controlled trials? A quasi-randomised, interview-based study. *Br Med J* **318**: 1114–1117

*© 2000 Cancer Research Campaign*

### Appendix 1: Accept and decline questionnaire

**CONFIDENTIAL** **ID..........**

### CLINICAL TRIALS QUESTIONNAIRE

We are interested in the reasons why patients **accept** or **decline** to take part in clinical trials/studies. We would be grateful if you would fill in this questionnaire. It will not be shown to your doctor or any of the staff at the hospital. A pre-paid envelope is provided for the return of the form.

| | Yes | No | Do Not Know |
|---|---|---|---|
| First, we would like to know if you have agreed to take part in a clinical trial/study? | ☐ | ☐ | ☐ |

Below are some reasons that may have influenced your decision to **accept** or **decline** to take part in a clinical trial/study. Please answer each question and tick the box that shows most clearly how you feel.

| | **Strongly agree** | **Agree to some extent** | **Unsure** | **Disagree to some extent** | **Strongly disagree** |
|---|---|---|---|---|---|
| 1) I thought the trial/study offered the best treatment available. | ☐ | ☐ | ☐ | ☐ | ☐ |
| 2) I believed the benefits of treatment in the trial/study would out-weigh any side-effects. | ☐ | ☐ | ☐ | ☐ | ☐ |
| 3) I was satisfied that either treatment in the trial/study would be suitable for me. | ☐ | ☐ | ☐ | ☐ | ☐ |
| 4) I was worried that my illness would get worse unless I joined the trial/study. | ☐ | ☐ | ☐ | ☐ | ☐ |
| 5) The idea of randomisation worried me. | ☐ | ☐ | ☐ | ☐ | ☐ |
| 6) I wanted the doctor to choose my treatment rather than be randomised by computer. | ☐ | ☐ | ☐ | ☐ | ☐ |
| 7) The doctor told me what I needed to know about the trial. | ☐ | ☐ | ☐ | ☐ | ☐ |
| 8) I trusted the doctor treating me. | ☐ | ☐ | ☐ | ☐ | ☐ |
| 9) I was given too much information to read about the trial | ☐ | ☐ | ☐ | ☐ | ☐ |
| 10) I was given enough information to read about the trial | ☐ | ☐ | ☐ | ☐ | ☐ |
| 11) I knew that I could leave the trial/study at any time and still be treated. | ☐ | ☐ | ☐ | ☐ | ☐ |
| 12) I did not feel able to say no. | ☐ | ☐ | ☐ | ☐ | ☐ |
| 13) I wanted to help with the doctors research. | ☐ | ☐ | ☐ | ☐ | ☐ |
| 14) I feel that others with my illness will benefit from the results of the trial. | ☐ | ☐ | ☐ | ☐ | ☐ |
| 15) The doctor wanted me to join the trial/study. | ☐ | ☐ | ☐ | ☐ | ☐ |
| 16) Others, e.g. family or friends wanted me to join the trial/study. | ☐ | ☐ | ☐ | ☐ | ☐ |

Which was the most important reason for you out of the list? (Please give number)____________

*© 2000 Cancer Research Campaign*
